# IκB Kinase β Regulates Epithelium Migration during Corneal Wound Healing

**DOI:** 10.1371/journal.pone.0016132

**Published:** 2011-01-17

**Authors:** Liang Chen, Qinghang Meng, Winston Kao, Ying Xia

**Affiliations:** 1 Department of Environmental Health, University of Cincinnati Medical Center, Cincinnati, Ohio, United States of America; 2 Department of Ophthalmology, University of Cincinnati Medical Center, Cincinnati, Ohio, United States of America; University of Reading, United Kingdom

## Abstract

The IKKβ is known to regulate transcription factor NF-κB activation leading to inflammatory responses. Recent gene knockout studies have shown that IKKβ can orchestrate local inflammatory responses and regulate homeostasis of epithelial tissues. To investigate whether IKKβ has an intrinsic role in epithelial cells, we established an *in vivo* system in the immune privileged corneal epithelium. We generated triple transgenic *Krt12^rtTA/rtTAt^/tet-O-Cre/Ikkβ^F/F^* (*Ikkβ^ΔCE/ΔCE^*) mice by crossing the *Krt12*-rtTA knock-in mice, which express the reverse tetracycline transcription activator in corneal epithelial cells, with the *tet-O-Cre* and *Ikkβ^F/F^* mice. Doxcycline-induced IKKβ ablation occurred in corneal epithelial cells of triple transgenic *Ikkβ^ΔCE/ΔCE^* mice, but loss of IKKβ did not cause ocular abnormalities in fetal development and postnatal maintenance. Instead, loss of IKKβ significantly delayed healing of corneal epithelial debridement without affecting cell proliferation, apoptosis or macrophage infiltration. In vitro studies with human corneal epithelial cells (HCEpi) also showed that IKKβ was required for cytokine-induced cell migration and wound closure but was dispensable for cell proliferation. In both in vivo and in vitro settings, IKKβ was required for optimal activation of NF-κB and p38 signaling in corneal epithelial cells, and p38 activation is likely mediated through formation of an IKKβ-p38 protein complex. Thus, our studies in corneal epithelium reveal a previously un-recognized role for IKKβ in the control of epithelial cell motility and wound healing.

## Introduction

The IκB kinase (IKK) complex, composed of two kinases (IKKα and IKKβ) and a regulatory subunit IKKγ, is the critical signaling mediator for classical NF-κB activation [1], [2]. Diverse stimuli, including injury, infection, inflammation and environmental stresses, such as UV-irradiation, can activate IKK [3]. Once activated, the IKK complex, especially the IKKβ subunit, is responsible for catalyzing IκB phosphorylation, leading to a rapid IκBα ubiquitination and degradation. This results in the release of the nuclear factor-κB (NF-κB) transcription factor, which in turn translocate to the nucleus, bind to DNA and activate gene transcription. Through this well-established paradigm, the IKKβ-NF-κB signaling pathways lead to rapid reprogramming of gene expression in essentially all mammalian cell types [4].

The IKKβ is best known for mediating activation of the classical NF-κB cascades by pro-inflammatory cytokines and pathogen-associated molecular patterns (PAMPs) and is instrumental for regulating innate immunity and inflammatory responses [3]. However, recent findings in gene-targeted mice suggest broader implications of IKKβ in the maintenance of homeostasis, stress responses and regulation of survival and apoptosis. While systematic *Ikkβ* gene deletion in mice leads to embryonic lethality [5], [6], conditional *Ikkβ* ablation in specific cell types has largely avoided developmental defects. Studies of these mice so far reveal diverse cell type-specific roles of IKKβ. In keratinocytes, IKKβ acts to maintain the immune homeostasis of the skin [7], [8]; in neurons, it inhibits sensory neuron excitability [9]; in hepatocytes, it suppresses cell proliferation [10], [11]; and in mammary epithelial cells, IKKβ potentiates apoptosis that leads to mammary gland involution [12].

Studies on knockout mice also strongly suggest that IKKβ has dual protective and destructive roles in response to injury and environmental insults. While IKKβ is pro-apoptotic in germ cells responding to ionizing radiation [13], it is anti-apoptotic in intestinal and gastric epithelial cells responding to bacterial infection and burn [14], [15], [16]. Moreover, IKKβ has anti-apoptotic roles in protection of cardiomyocytes from pressure overload [17] and of osteoclasts from cytokine-induced apoptosis [18]. The in vivo roles of IKKβ depend not only on the IKKβ-mediated specific cell response, but also on its ability to modulate inflammatory crosstalk in the surrounding environment. For example, protection of host intestinal tract from bacterial infection by the intestinal epithelial IKKβ is the consequence of both reduced neutrophil infiltration that suppresses local inflammation and increased epithelial cell survival [16]. The hepatocyte IKKβ prevents chemical carcinogenicity by alleviating the activation of liver macrophage, which produces mitogens that drive the compensatory hepatocyte proliferation, and reducing hepatocyte ROS accumulation and apoptosis [19]. Hence, the diverse roles displayed by IKKβ in vivo are attributed to the combined effects on specific cell activities and local inflammatory responses.

The cornea of the eye consists of five distinct layers: a stratified non-keratinized epithelial cell layer, the Bowman's membrance, a highly organized collagenous stroma layer interspersed with keratocytes, the Descemet's membrane and a single endothelial cell layer [20]. Among these, the corneal epithelium is the outermost layer and therefore it is readily exposed to various external insults and is particularly susceptible to injuries caused by trauma, infections and thermal or chemical exposure [21], [22]. A simple corneal epithelium debridement injury causes minor disruption of the underlying stroma and elicits only slight inflammation, and the healing depends primarily on corneal epithelial cell activities, including migration, proliferation and re-stratification [23], [24]. For these reasons, the corneal epithelial debridement is widely used as a tool to assess the epithelial cell responses to injury in experimental settings.

Though corneal epithelial injury usually does not elicit severe inflammatory cell infiltration in the wounding areas, it induces mild inflammatory cytokine responses [22], [24], [25]. A number of in vitro studies suggest that the cytokine response can promote re-epithelialization and assist healing, but the molecular and signaling basis has remained largely uncharacterized [26], [27], [28]. In the present studies, we generated triple transgenic mice *Krt12^rtTA/rtTA^/tet-O-Cre/Ikkβ^F/F^*, in which the *Ikkβ* gene is ablated specifically in corneal epithelial cells when fed with doxycycline. We used these mice to investigate the roles of IKKβ, a key transducer of cytokine signaling, in corneal epithelial wound healing in vivo. In addition, we used human telomerase-immortalized corneal epithelial (hTCEpi) cells and human keratinocytes (HaCaT) to identify the cellular and signaling properties of IKKβ in vitro. Our studies have identified a previously unrecognized role of IKKβ in potentiating epithelial cell migration and wound healing through the activation of NF-κB and p38 cascades.

## Results

### IKKβ is dispensable for development and homeostasis of the corneal epithelium

**Figure 1:**
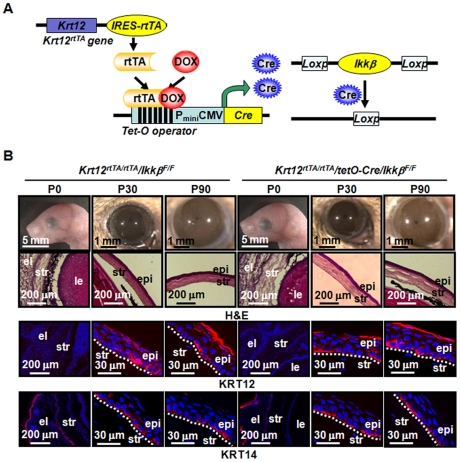
Genetic deletion of *Ikkβ* gene has no effect on the developmental morphogenesis of mouse corneal epithelium. (A) An illustration of the triple transgenic mice (*Krt12^rtTA/rtTA^/tet-O-Cre/Ikkβ^F/F^*), consisting of (1) corneal epithelial-specific keratin 12 promoter driven *rtTA*, (2) a *tet-pCMV-O-cre* allele and (3) the *Ikkβ^F/F^* alleles. The doxycycline can activate rtTA to induce the Cre expression in corneal epithelial cells, and Cre in turn mediates the *Ikkβ* gene ablation. (B) The eyes of double (*Krt12^rtTA/rtTA^/Ikkβ^F/F^*, *Ikkβ^F/F^*) and triple (*Krt12^rtTA/rtTA^/tetO-Cre/Ikkβ^F/F^*, *Ikkβ^ΔCE/ΔCE^*) transgenic littermates exposed to doxycycline in embryonic stages were photographed at 0, 30 and 90 days after birth. The eye sections were examined histologically by H&E staining and immunohistochemically for the expression of KRT12 and KRT14 (red). Nuclei were stained with DAPI (blue). Pictures represent at least 3 slides of each mouse and 2 mice of each genotype were examined. el: eyelid, str: corneal stroma, epi: corneal epithelium, le: lens, and dotted lines mark the boundary between corneal epithelium and stroma.

Previously, our laboratories used a targeted knock-in strategy to generate the *Krt12^rtTA^* mouse line. This transgenic mouse produced a bicistronic mRNA coding for both cytokeratin 12 (KRT12) and reverse tetracycline transcription activator (rtTA) under the control of the corneal epithelium-specific *Krt12* promoter, which is activated as early as embryonic day 14.5. The *Krt12^rtTA^/tet-O-Cre* system has been previously used to generate mouse lines for inducible gene ablation in a corneal epithelium-specific manner [29]. Using these resources, we made the *Krt12^rtTA/rtTA^/tet-O-Cre/Ikkβ^F/F^* triple transgenic mice, which could be induced for corneal epithelium-specific *Ikkβ* gene ablation (Fig. 1A).

To evaluate whether IKKβ is required for corneal epithelium morphogenesis during development, we fed the females with doxycycline chow (DOX-chow), starting from the date of conception until weaning of the offspring. The *Krt12^rtTA/rtTA^/Ikkβ^F/F^* and *Krt12^rtTA/rtTA^/tetO-Cre/Ikkβ^F/F^* offspring were kept in Dox-chow and their eyes were examined at 0, 30 and 90 days after birth (Fig. 1B). Regardless of the genotypes, all of the offspring had normal appearance of the eyes, with no abnormalities in the size of the eye, and the thickness and transparence of cornea. They also had identical normal histological features, with the same expression pattern of KRT12 and cytokeratin 14 (KRT14), epithelial differentiation markers. While KRT12 expression was absent in many basal cells in young mice, it was detected in suprabasal and superficial epithelial cells at 30 and 90 days after birth. On the other hand, KRT14 was mainly expressed in the basal epithelial cells at all stages observed. These results suggest that IKKβ is dispensable for normal development, morphogenesis and differentiation of the corneal epithelium.

**Figure 2:**
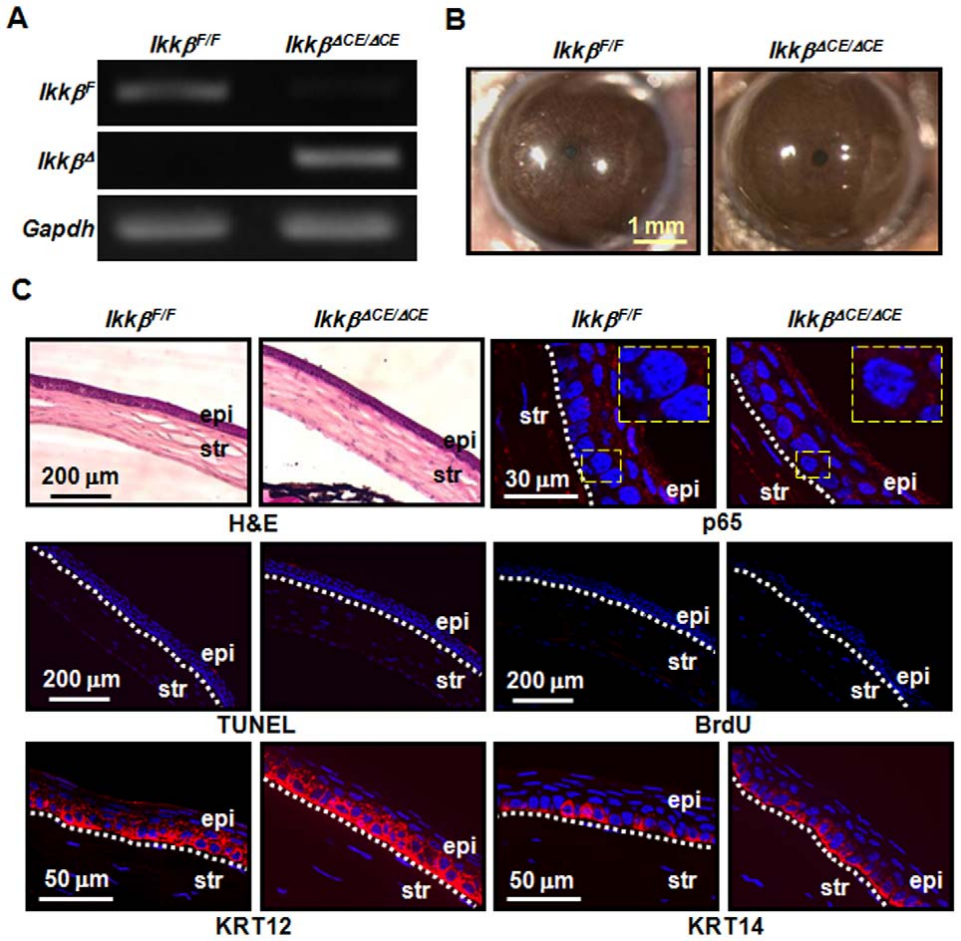
IKKβ is dispensable for the maintenance of mouse corneal epithelium. The adult double (*Ikkβ^F/F^*) and triple (*Ikkβ^ΔCE/ΔCE^*) transgenic mice were fed with Dox-chow for 30 days. (A) Genomic DNA was isolated from the corneal epithelial cells and subjected to PCR genotyping using primers specific for the *Ikkβ^Δ^* (deleted allele), *Ikkβ^F^* (floxed allele) and *Gapdh*. The triple transgenic mice lost *Ikkβ^F^* and acquired *Ikkβ^Δ^* alleles. (B) The eyes were photographed and (C) their tissue sections were subjected to histological analyses by H&E staining and molecular characterization by immunostaining (red) and nuclei were identified by DAPI staining (blue). The TUNEL assay was used to detect apoptosis, BrdU incorporation was used for proliferation, and the expression of KRT12 and KRT14 was used to evaluate corneal (KRT12) and basal (KRT14) epithelial differentiation. The activation of the IKK pathway was evaluated by p65 nuclear translocation and pictures were taken at low and high (rectangle inserts) magnifications. The boundaries of corneal epithelium (epi) and stroma (str) were marked with dotted lines. The picture represents at least 3 slides of each mouse and 2 mice of each genotype used for the studies.

To evaluate the roles of IKKβ in maintenance of corneal homeostasis, we fed the adult double (*Krt12^rtTA/rtTA^/Ikkβ^F/F^*) and triple (*Krt12^rtTA/rtTA^/tetO-Cre/Ikkβ^F/F^*) transgenic mice Dox-chow for 30 days. To confirm the induction of *Ikkβ* gene deletion, we examined the genomic DNA isolated from corneal epithelial cells. By PCR, we detected only the *Ikkβ^F^* allele in cells isolated from double transgenic mice, whereas, we found only the *Ikkβ^Δ^* allele in cells isolated from triple transgenic mice (Fig. 2A). The triple transgenic mice with induced corneal epithelium-specific *Ikkβ* ablation are hereafter referred to as *Ikkβ^ΔCE/ΔCE^*, whereas the control double transgenic mice are referred to as *Ikkβ^F/F^*.

Examination using a stereo-microscope showed that the eyes of both *Ikkβ^F/F^* and *Ikkβ^ΔCE/ΔCE^* mice had normal appearance, with no abrasion, ulceration or haze of the cornea (Fig. 2B). Histological examination also revealed normal thickness and morphology of the cornea in both mice (Fig. 2C). The corneal epithelial homeostasis requires dynamic self-renew, involving the basal cell proliferation, migration upward and differentiation to suprabasal and superficial layer, which eventually sheds off [30]. We found that the corneal epithelium in both genotypes had low proliferation, no apoptosis and adequate expression patterns of differentiation markers, KRT12 and KRT14. We also observed that the NF-κB subunit p65 was located solely in the cytoplasmic compartment of corneal epithelial cells in the *Ikkβ^F/F^* mice, similar to that in the *Ikkβ^ΔCE/ΔCE^* mice, suggesting that the IKKβ-NF-κB pathway was mostly inactive in the corneal epithelial cells under normal physiological conditions devoid of stress and injury. Thus, loss of IKKβ does not seem to perturb homeostatic maintenance of the corneal epithelium in adult mice.

### The IKKβ is required for optimal corneal re-epithelialization

**Figure 3:**
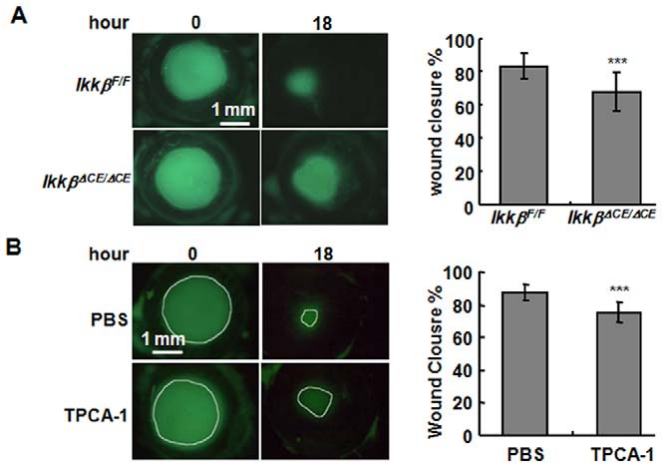
IKKβ is required for optimal re-epithelialization of corneal wounds in vivo. (A) The *Ikkβ^F/F^* and *Ikkβ^ΔCE/ΔCE^* mice and (B) the C57BL/6 adult mice were subjected to corneal epithelial debridement injury, and some mice were treated with TPCA-1 (5 µM) after injury as indicated. The wounded eyes were stained with green fluorescein for photography at 0 and 18 hours and the wound closure rates were calculated by comparing the wound areas at 18 versus 0 hours. The results are mean ± SD from 18 eyes (9 mice) of each genotype in (A), and from 6 eyes with or without TPCA-1 treatment in (B). Statistical analyses were performed and ***: *p<*0.001 is considered significant.

To determine whether IKKβ was required for stress response of the corneal epithelial cells, we introduced corneal epithelial debridement injuries to the *Ikkβ^F/F^* and *Ikkβ^ΔCE/ΔCE^* mice and examined the healing processes. We found that the *Ikkβ^ΔCE/ΔCE^* mice had clearly a larger wound remained than the *Ikkβ^F/F^* mice at 18 hours after injury (Fig. 3A), suggesting that IKKβ was required for optimal re-epithelialization. To confirm the findings made in the *Ikkβ^ΔCE/ΔCE^* mice, we examined the corneal epithelial injury in C57BL/6 mice treated with TPCA-1, a chemical inhibitor of IKKβ. Corneal epithelial debridement was generated on C57BL/6 mice, followed by topical application of either vehicle PBS or TPCA-1 at the wounded corneas. By 18 hours after injury, the epithelial wounds were reduced by 90% in the control PBS-treated corneas, similar to that of the *Ikkβ^F/F^* corneas (Figs. 3A and 3B); however, a larger wound was seen in the TPCA-1 treated mice, mimicking that in the *Ikkβ^ΔCE/ΔCE^* mice.

After injury, the damaged corneal epithelium usually regenerates quickly by a migration phase to cover the denuded area followed by a proliferative phase to obtain the normal epithelial thickness [24]. We showed that the *Ikkβ^F/F^* and *Ikkβ^ΔCE/ΔCE^* mice were almost identical at both the migration edge and peripheral corneas (Fig. S1). Both corneas had relatively quiescent basal epithelial cell proliferation around the wounding edge at the migration stage (6 hours and 18 hours), agreeing with the concept that the compensatory proliferation of corneal epithelial cells starts when re-epthelialization was almost ceased [24], [25]. Both corneas had readily detectable apoptosis induction and few macrophage (F4/80) accumulation in the stroma underneath the damaged epithelium, all shown previously as typical wound healing responses [22], [31]. Some TUNEL positive cells were seen in the stroma and endothelium distal to the injury sites (Fig. S1), supporting the idea that corneal epithelial injury can transactivate stromal cell apoptosis [22], [31]. Taken together, our results indicate that loss of IKKβ in corneal epithelial cells delays re-epithelialization by mechanisms independent of proliferation, apoptosis and macrophage activation.

### Induction of epithelial cell migration by inflammatory cytokines is mediated by IKKβ

**Figure 4:**
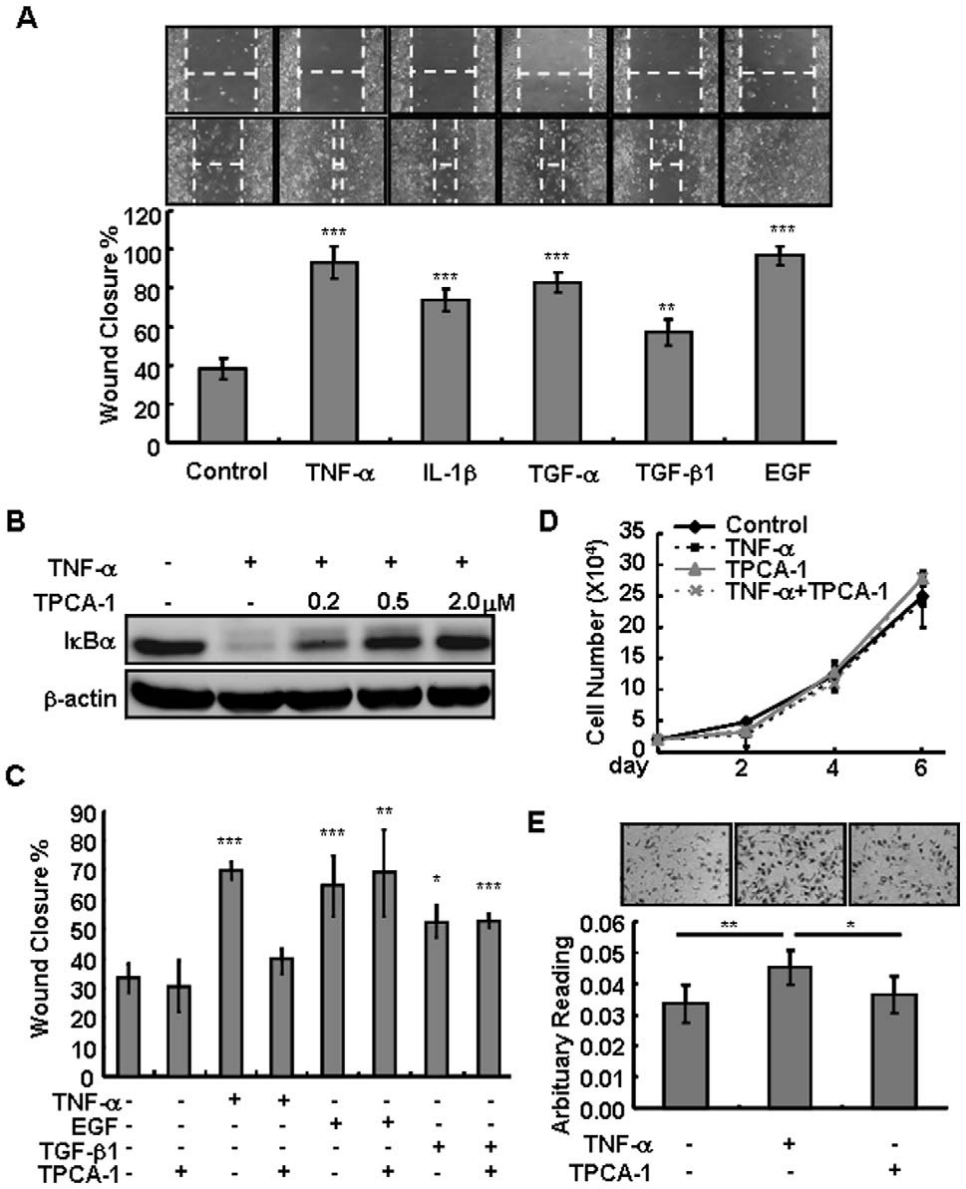
Cytokine stimulated hTCEpi cell wound closure is mediated through IKKβ. The hTCEpi cells were treated with various growth factors and cytokines (10 ng/ml) in the presence or absence of pre-treated with TPAC-1 at 0.5 µM for 0.5 hour or as indicated. (A and C) The cells were subjected to *in vitro* would healing assays. Pictures were taken at 0 and 48 hours and the wound closure rates were calculated by comparing the width of wounds at the beginning and end of the experiment. Results were shown as mean ± SD of 4 repeats. (B) Cell lysates were subjected to Western blotting for IκBα and β-actin. (D) The number of hTCEpi cells was counted at different time points of treatment and the cell growth curves under each condition were generated. The results represent two independent experiments. (E) Twenty-four hours after TNF-α treatment, the cells were subjected to trasnwell migration assay for 3 hours. The migrated cells were stained by crystal violet and photographed. Relative cell migration was quantified by measuring the absorbance of crystal violet dye. Results represent means ± SD of 4 independent experiments. Statistical analyses were performed and *: *p<*0.05; **: *p<*0.01; ***: *p<*0.001 were considered significant.

Corneal re-epithelialization is controlled by a number of growth factors/cytokines produced upon epithelial injury [21], [22], [24], [27], [30], [31]. We examined the effects of exogenous growth factors/cytokines on re-epithelialization of the human corneal epithelial hTCEpi cells using an in vitro scratch wound healing assay. When added to the scratch wounds created on hTCEpi cells, all the factors tested, including TNF-α, IL-1β, TGF-α, TGF-β1 and EGF, were able to potentiate wound closure, with TNF-α and EGF being the most efficient (Fig. 4A). Pre-treatment of the hTCEpi cells with TPCA-1 markedly blocked TNF-α-induced IκBα degradation thereby NF-κB activation (Figs. 4B). Interestingly, TPCA-1 significantly prevented wound closure induced by TNF-α and IL-1β, but had little, if any, effect on wound closure induced by EGF and TGF-β1 (Figs. 4C and S2A). The in vitro wound healing is a coordinated process involving epithelial cell proliferation and migration. We found that neither TNF-α nor TPCA-1 was able to alter the hTCEpi cell proliferation; however, TNF-α significantly potentiated the cell motility, which was abolished by TPCA-1 (Figs. 4D and 4E). Our results strongly suggest that IKKβ is required for inflammatory cytokines to stimulate corneal epithelial cell migration and wound closure.

To determine whether the cytokine-IKKβ axis was effective in promoting wound closure of other cell types, we examined human epidermal epithelial HaCaT cells. Similar to that of hTCEpi, the wound closure of HaCaT cells was significantly induced by TNF-α, IL-1β, EGF, and TGF-β1, and the induction by TNF-α, but not TGF-β1, was inhibited by TPCA-1 (Figs. S2B and S2C). In contrast, the wound closure of HTKs, a telomerase-immortalized human corneal fibroblasts [32], and murine embryonic fibroblasts (MEFs) was unaffected by TNF-α (data not shown), suggesting that the cytokine-IKKβ axis is involved in wound closure of epithelial cells, but not fibroblasts.

### IKKβ is responsible for cytokine-induced activation of the p65 and p38/ATF2 cascades in hTCEpi cells

**Figure 5:**
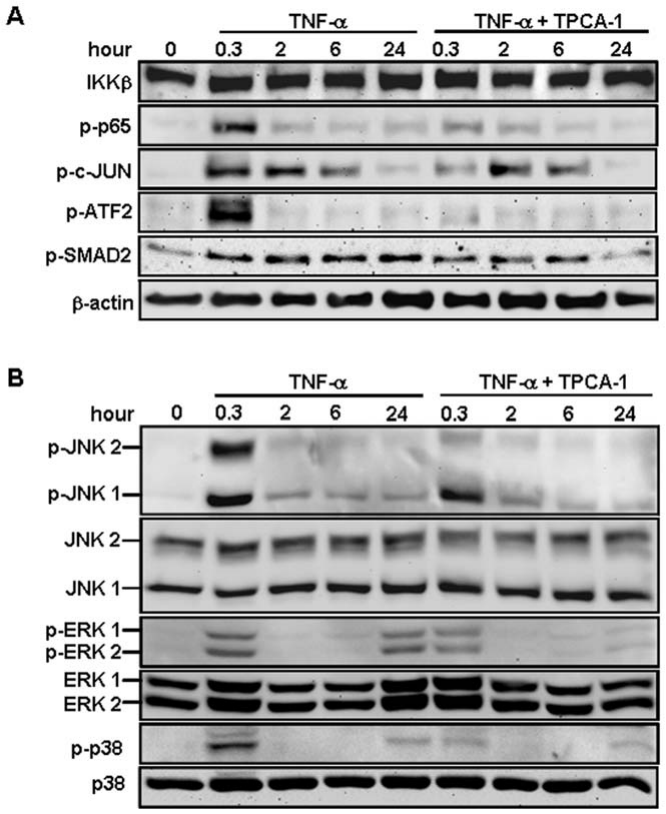
Inhibition of IKKβ prevents TNF-α induced activation of NF-κB, p38 andATF2. The hTCEpi cells were treated with TNF-α (10 ng/ml) for indicated times in the presence or absence of TPCA-1 (0.5 µM). Cell lysates were analyzed by Western blotting to detect (A) the expression level of IKKβ and β-actin, and the phosphorylation of p65, c-JUN, ATF2 and SMAD2, and (B) the phosphorylation and expression of JNK, ERK and p38. Induction of p65, p-ATF2 and p-p38 by TNF-α is significantly blocked by TPCA-1. Pictures were representative of at least 2 repeated experiments.

Corneal epithelial wound healing is orchestrated by cytokines, which activate various signaling pathways, such as p38, JNK, ERK and TGF-β/SMAD [22], [33], [34], [35], [36]. To identify the signaling events downstream of IKKβ, we characterized the phosphorylation/activation of transcription factors and signaling kinases in the hTCEpi cells treated with TNF-α and TPCA-1. TNF-α caused an immediate but transient phosphorylation of p65, ATF2, JNK and p38; it induced an immediate and persistent phosphorylation of c-JUN and SMAD2, and an immediate and delayed activation of ERK (Figs. 5A and 5B). The delayed ERK activation is likely due to transcriptional activation of early response genes that in turn reactivate the same pathway at later stage, as reported before [37]. Interestingly, TPCA-1 pre-treatment inhibited only the phosphorylation of p65 and p38/ATF2, but had little effect on other signaling events.

**Figure 6:**
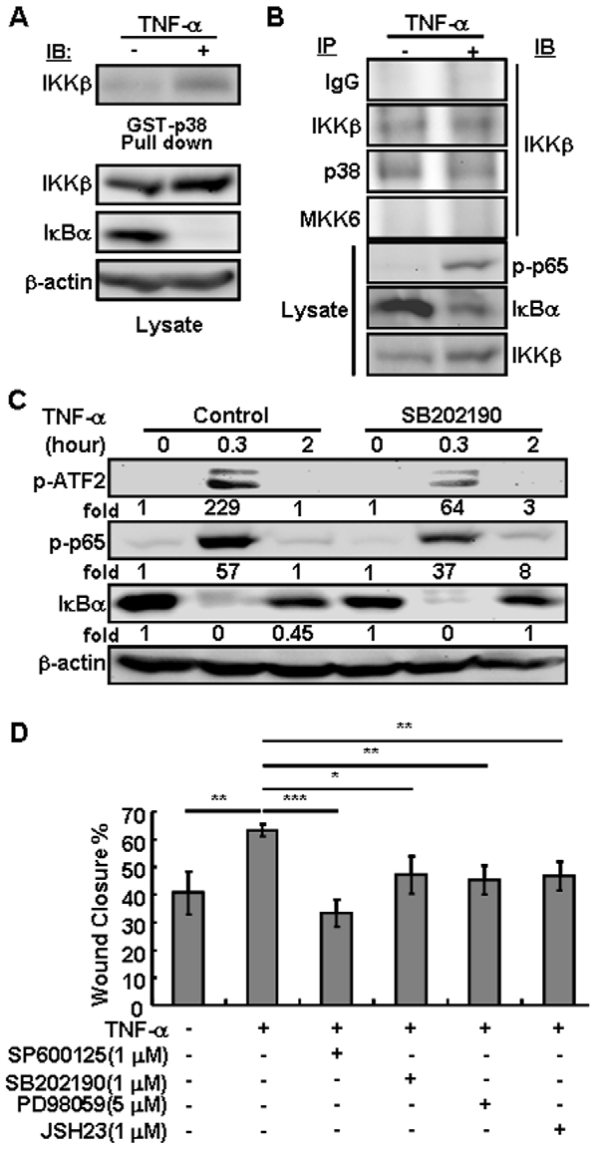
The IKKβ and p38 form complexes in vitro and in vivo. The hTCEpi cells were either untreated or treated with TNF-α (10 ng/ml) for 0.5 hour or as indicated. In some experiments, chemical inhibitors for p38, SB202190 (1 µM), JNK, SP600125 (1 µM), ERK, PD98059 (5 µM), and IKK, JSH23 (1 µM), were used 0.5 hour prior to TNF-α. Cell lysates were subjected to (A) pull-down assays using GST-p38 and glutathione-agarose beads, and (B) immunoprecipitation using anti-p38, anti-IKKβ and anti-MKK6 antibodies. The pull-down/precipitated proteins and total cell lysates were analyzed by Western blotting using antibodies as indicated. (C) The cell lysates were analyzed by Western blotting for phospho-ATF2 and -p65, and total IκBα and β-Actin. The relative fold induction was calculated based on the intensity of the bands in control (set as 1) and TNF-α treated samples. (D) The hTCEpi cells were either untreated or pre-treated with various chemical inhibitors as indicated for 0.5 hour. The cells were subjected to in vitro scratch wound healing assay in the presence or absence of TNF-α (10 ng/ml) for 48 hours. The wound closure rates were calculated based on the mean ± SD of 4 repeats and statistical analyses were done by comparing to the rates in control cells. *: *p<*0.05; **: *p<*0.01; ***: *p<*0.001.

The molecular connection of IKKβ to NF-κB, based on direct interaction and phosphorylation of IκBα, is well established, but the link to p38 remains obscure. To look into the molecular basis of the latter, we examined the physical interactions between IKKβ and p38. The hTCEpi cells were either un-treated or treated with TNF-α for 20 min to induce an apparent IκBα degradation and p65 phosphorylation, indicative of the NF-κB pathway activation (Figs. 6A and 6B). From both un-treated and TNF-α-treated hTCEpi cells, the GST-p38 and anti-p38 antibodies were able to pull down IKKβ (Figs. 6A and 6B). The p38 is a mitogen-activated protein kinase, known to interact with and be phosphorylated by its upstream kinases, MKK3, MKK4 and MKK6, in response to mitogenic and stress stimuli [38]. Antibodies to MKK6, however, were unable to co-precipitate IKKβ, indicating that the IKKβ-p38 complexes were independent of MKK6. We suggest that the IKKβ-p38 complexes are distinct from the MKK6-p38 and are used primarily for effective and specific transduction of cytokine signals in hTCEpi cells (Fig. 5).

It is possible that the IKKβ-p38 interaction allows p38 to act upstream of IKKβ, responsible for activation of NF-κB [39]. To test this possibility, we used a p38 inhibitor SB202190 to pre-treat the hTCEpi cells prior to TNF-α exposure. While the inhibitor caused a significant 75% decrease of ATF2 phosphorylation, it did not affect the induction of IκBα degradation and p65 phosphorylation by TNF-α (Fig. 6C). In contrast, the IKKβ inhibitor significantly prevented p38 activation (Fig. 5), supporting the idea that IKKβ acts upstream to activate the p38-ATF2, in addition to activate the NF-κB pathways, in hTCEpi cells.

To evaluate the contributions of the downstream signaling events to wound closure, we pre-treated the hTCEpi cells with specific inhibitors of individual pathways before making scratch wounds. We found that the induction of wound closure by TNF-α was significantly blocked not only by inhibitors of p38 and NF-κB, but also by inhibitors of JNK and ERK (Fig. 6D). Hence, TNF-α induced corneal epithelial wound healing requires both IKKβ-dependent and -independent pathways.

### IKKβ is required for activation of the p65 and p38/ATF2 cascades in injured corneal epithelium

**Figure 7:**
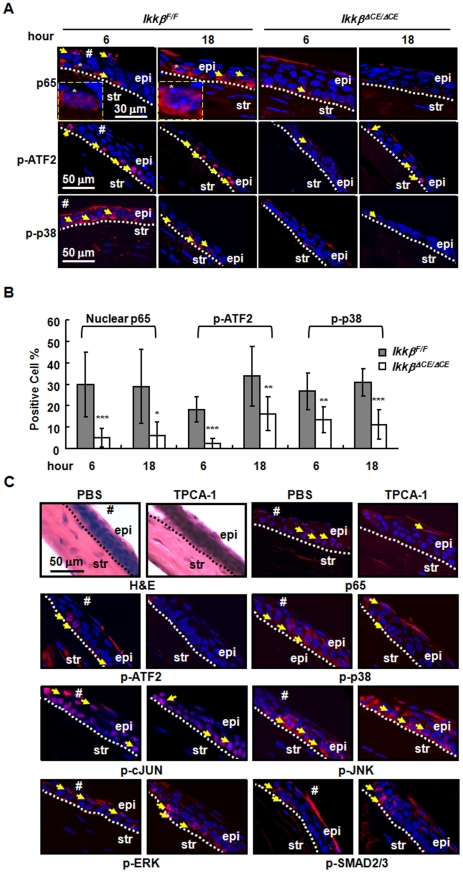
Injury-induced p65 and p38/ATF2 activation in corneal epithelium depends on IKKβ. The *Ikkβ^F/F^* and *Ikkβ^ΔCE/ΔCE^* mice were subjected to corneal epithelial debridement injury and the wounded eyes were examined by immunohistochemistry for p65, p-ATF2 and p-p38 (red) and nuclei were labeled by DAPI (blue). (A) Pictures were taken and the photographs represented at least 3 slides of 2 eye samples. The nuclear p65, p-ATF2 and p-p38 positive cells can be identified in the injured corneal epithelium (arrows) and the nuclear location of p65 (*) is clearly visible in pictures under higher magnification (dotted squares). (B) The percentages of corneal epithelial cells that were staining positive in each field were calculated. At least 3 sections of each eye and 2 eyes of each genotype at a given time point were used for calculation and statistical analysis. *: *p<*0.05; **: *p<*0.01; ***: *p<*0.001 were considered significant different between *Ikkβ^F/F^* and *Ikkβ^ΔCE/ΔCE^* mice. (C) C57BL/6 adult mice were subjected to corneal epithelial debridement injury, followed by application of PBS to one eye and TPCA-1 (5 µM) to the other eye for 2 hours. At 6 hours after wounding, the eyes were collected and analyzed by H&E staining and by immunohistochemistry using antibodies as indicated. Cells positive for nuclear translocation of p65 and phospho-ATF2, -p38, -c-JUN, -JNK, -ERK and -SMAD2/3 were pointed with arrows. Pictures were chosen from at least 3 slides of each eye and 2 eyes of each genotype used. str: corneal stroma, epi: corneal epithelium. Dotted lines mark the boundary between corneal epithelium and stroma, and # marks the edge of the corneal wound.

The in vitro studies in hTCEpi cells have identified several IKKβ-dependent and -independent signaling events in response to inflammatory cytokine TNF-α (Fig. 5). To determine the signaling properties of IKKβ in vivo, we examined the healing eyes of *Ikkβ* ^F/F^ and *Ikkβ^ΔCE/ΔCE^* mice. We found that IKKβ was required for the induction of p65 nuclear translocation and phosphorylation of the p38 MAPK and its downstream transcription factor ATF2 (Figs. 7A and 7B), but was dispensable for the activation of JNK, c-JUN, ERK and SMAD pathways (Fig. S3). While approximately 30–40% corneal epithelial cells adjacent to the wounding area were stained positive for nuclear p65 and phospho-p38 and -ATF2 in the *Ikkβ^F/F^* mice, significantly fewer cells were stained positive in the *Ikkβ^ΔCE/ΔCE^* mice. These observations suggest that unlike its relatively quiescent state in the normal cornea, IKKβ appears to be activated in the corneal epithelial cells after injury.

We reached the same conclusion by comparison of the control and TPCA-1 treated corneas after epithelial debridement injury. Although control and TPCA-1 treated corneas had no obvious morphological differences, they had different patterns of signaling pathway activation. In the areas adjacent to the injury, the TPCA-1-treated corneas had significantly fewer epithelial cells that were stained positive for nuclear p65, phosphor-p38 and phosphor-ATF2, whereas, the phosphorylation of c-JUN, JNK, ERK and SMAD2/3 were detected at the similar levels in control and TPCA-1 treated corneas (Fig. 7C). Thus, inhibition of IKKβ by genetic and pharmacological means both prevented or reduced activation of the NF-κB and p38-ATF2 cascades in the injured corneal epithelium.

## Discussion

Using the corneal epithelial debridement model and mice with inducible and cell type-specific *Ikkβ* gene ablation, we have shown that the corneal epithelial IKKβ is required for optimal re-epithelialization and wound healing. While loss of IKKβ does not seem to perturb the injury-induced proliferation, apoptosis and macrophage activation, it significantly reduces the activation of NF-κβB and p38/ATF2 pathways in the corneal epithelial cells adjacent to the injury sites. Correspondingly, in cultured hTCEpi human corneal epithelial cells IKKβ is required for activation of NF-κB and p38/ATF2, and induction of cell migration and wound closure by inflammatory cytokines. Because the IKKβ-mediated signaling events in injured corneal epithelium are strikingly similar to those in TNF-α treated hTCEpi cells, we suggest that injury may induce inflammatory cytokine to activate the IKKβ signaling cascades, which in turn play a predominant role in promoting corneal re-epithelialization in vivo.

Similar to its roles in hTCEpi cells, IKKβ promotes wound healing of epidermal keratinocytes HaCaT, but not corneal stromal fibroblasts HTK and embryonic fibroblasts. These observations suggest that IKKβ has a unique role in promoting the migration of epithelial cells, a conclusion supported only by the *in vivo* corneal wound healing model, but not by other models where IKKβ ablation results in complications in the local environment. For example, IKKβ ablation in the skin epidermis leads to auto-immune diseases of the skin, precluding the use of the knockout mice to study wound healing [7], whereas, IKKβ ablation in intestinal epithelium results in aberrant epithelial cell survival and proliferation in a number of injury models [15], [40]. The corneal epithelial IKKβ is unique in that it is inactive in the naive, uninjured cornea and is dispensable for the developmental morphogenesis and homeostatic maintenance of mouse corneas. Yet, it is activated by corneal epithelial debridement injury, which affects predominantly the surface epithelial cells, but has little influences on the underneath stroma and does not elicit severe inflammation due to the immune privileged nature of the cornea [41]. Thus, the unique features of the experimental system allow the identification of a novel role of IKKβ in controlling epithelial cell migration during wound healing.

Previous in vitro studies have showed that IKKβ acts through NF-κB-dependent and -independent mechanisms to mediate cytokine induced migration of normal and cancer cells [42], [43], [44], [45]. Specifically, active IKKβ can directly stimulate the phosphorylation of docking protein 1 (Dok1), the ras-GTPase-activating protein-associated tyrosine kinase substrate, to promote cell motility [42]; it can stabilize the transcriptional repressor Snail that leads to cell migration and invasion [44]; and it also can up-regulate the expression of matrix metalloproteinase-9, which in turn promotes extracellular matrix remodeling and migration [43]. Our data point at the specific roles of corneal epithelial IKKβ in the optimal activation of NF-κB and p38, but is dispensable for the activation of JNK, ERK and SMAD cascades, which are also induced by cytokines and corneal epithelial injuries. The activation of p38 by pro-inflammatory stimuli, such as LPS, has been shown previously mediated through the upstream TAK1-MKK3/6 cascades in lymphocytes and lung epithelial cells [46], [47], [48]. We find that IKKβ forms complexes with p38, but not MKK6, suggesting the existence of a distinct IKKβ-p38 signaling module in corneal epithelial cells. In this module, activation of p38 by IKKβ can be direct, or it can be mediated through TAK1 acting upon the MKK3/MKK4. Given that IKKβ inhibition significantly reduces but does not completely abolish p38 activation, we suggest both IKKβ-dependent and -independent mechanisms exist in corneal epithelial cells for p38 activation [38]. Activation of p38-ATF2 cascades in turn may regulate gene expression to contribute to migration of corneal epithelial cells, as well as of epidermal and mammary epithelial cells [33], [49], [50], [51].

It is interesting to note that corneal epithelial injury triggers the local release of a number of growth factors and cytokines, but only the pro-inflammatory cytokines need IKKβ to promote epithelial cell migration and re-epithelialization of corneal wounds [22], [24], [52]. Other cellular activities, such as survival and proliferation also essential for successful wound healing, seem to be regulated by IKKβ-independent signaling events [22], [24], [52]. A prompt healing of corneal surface wound is vital to maintain corneal transparency and preserve normal vision. In this regard, identification of the roles IKKβ play in epithelial cell migration and wound healing is of great clinical significance. This is because a number of corticosteroid and nonsteroidal anti-inflammatory drugs (NSAID) commonly used to alleviate pain after surgery may act by inhibition of the IKKβ signaling to cause delayed wound healing and persistent epithelial defects [53], [54], [55], [56], [57]. Thus, alternative therapeutics avoiding IKKβ inhibition may be more favorable for treating diseases that require prompt corneal epithelial wound healing.

## Materials and Methods

### Reagents, antibodies and cell culture

Cytokines and growth factors, including TNF-α, IL-1β, EGF, TGF-α, TGF-β1, were purchased from PeproTech Inc. (Rocky Hill, NJ). The chemical inhibitors for JNK (SP600125), p38 (SB202190), ERK (PD98059) were obtained from Calbiochem (Gibbstown, NJ); and the inhibitor for IKKβ (TPCA-1) was from Tocris Bioscience (Ellisville, Missouri). Antibodies for IKKβ, phospho-p38 (Thr-180, Tyr-182) and total p38, phospho-ERK (Thr-202, Thr-204) and total ERK, as well as antibodies for phospho-c-Jun (Ser-63, 73), phospho-ATF2 (Thr-69, 71), phospho-p65 (Ser-536), phospho-SMAD2 (Ser-465, 467) and phospho-SMAD3 (Ser-423, 425), were purchased from Cell Signaling Technology (Danvers, MA); Antibodies for total JNK, total MKK6 and total p65 were purchased from Santa Cruz Biotechnology (Santa Cruz, CA); Antibodies for phospho-JNK (Thr-183, Tyr-185) (Promega, Madison, WI), F4/80 (ABcam Inc, Cambridge, MA), IκBα (BD Biosciences, San Jose, CA), β-actin (Sigma-Aldrich, St. Louis, MO), BrdU (Termo Fisher Scientific Inc, Waltham, MA), KRT14 (Covance, Alice, TX) and KRT12 [58] were used as well.

The hTCEpi and HaCaT cells were maintained in Keratinocyte Serum Free Medium (Invitrogen Corp., Carlsbad, CA), supplemented with 25 µg/ml Bovine Pituitary Extract, 0.2 ng/ml EGF, 50 U/mL penicillin, 50 µg/mL streptomycin. Medium was changed every 2 days.

### Generation of transgenic mice

Experimental animals were housed at the Experimental Animal Laboratory at the University of Cincinnati and all animal protocols were approved by the Institutional Animal Care and Use Committee (IACUC) of the University of Cincinnati (protocol no. 06-04-19-01). C57BL/6 mice were purchased from the Jackson Laboratory (Bar Harbor, ME). Compound transgenic mice lines, *Krt12^rtTA/rtTA^/tetO-Cre/Ikkβ^F/F^* and *Krt12^rtTA/rtTA^/Ikkβ^F/F^*,were generated by crossing the *Krt12^rtTA/rtTA^/tetO-Cre* [29], [59] with *Ikkβ^F/F^* mouse lines [60]. *Krt12^rtTA/rtTA^/tetO-Cre/Ikkβ^F/F^* female and *Krt12^rtTA/rtTA^/Ikkβ^F/F^* male mice were further crossed and fed Dox-chow (1 g/kg chow, Bioserv Corp., San Diego, CA) differently according to different experimental purposes. Genotyping was performed by polymerase chain reaction (PCR) using oligonucleotide primers specific for each transgene and the genomic DNA extracted from tail clip or corneal epithelial cells scraped from mice.

### In vivo corneal epithelial debridement injury in mice and evaluation of healing rate

Before debridement injury, 3-month old mice were anaesthetized by intraperitoneal administration of Avertin at 0.45 mg/g body weight (2, 2, 2-tribromoethanol, Sigma-Aldrich). The central corneas of both mouse eyes were demarcated by the Miltex Disposable Biopsy Punche (2 mm in diameter, Integra, Plainsboro, NJ) and a round epithelial debridement (2 mm in diameter) was produced using the Algerbrush II Corneal Rust Ring Remover (Ambler Surgical Corp., Exton, PA). A drop of fluorescein dye (Fluorescein Sodium Ophthalmic Strips, Akorn Inc, Lake Forest, Illinois) was applied to the injured cornea and the eyes were examined by florescent microscopy at different time points following the debridement injury. The rate of wound healing was calculated by comparing the wound areas at 0 and 18 hours after injury. At 4 and 16 hours after injury, the mice were injected intraperitoneally with BrdU at 0.1 mg/g body weight (Sigma-Aldrich) and sacrificed 2 hours later by CO_2_ asphyxia and cervical dislocation. The eye balls were collected and fixed by 4% paraformaldehyde (Sigma-Aldrich) in PBS buffer (Invitrogen) overnight, followed by dehydration through a graded sucrose series and embedding in OCT compound (Sakura Finetek, Torrance, CA).

### Histological and immunohistochemical analysis

Cryosections (8 µm in thickness) of the eye tissues were stained with hematoxylin and eosin (H&E) according to standard procedures. Sections were also subjected to immunohistochemical analysis as described previously [61], using anti-phospho-JNK (1∶100), -phospho-p38 (1∶100), -phospho-ERK (1∶100), -phospho-c-JUN (1∶100), -phospho-ATF2 (1∶100), -phospho-Smad2 (1∶100), -phospho-Smad3 (1∶100), -p65 (1∶100), -F4/80 (1∶100), -KRT12 (1∶100), -KRT14 (1∶100) antibodies. Stained sections were mounted (Vectashield Mounting Medium, Vector Laboratories Inc., Burlingame, CA) and photographed using an Axio Observer Inverted Microscope (Carl Zeiss Microimaging Inc., Thornwood, NY).

### In vivo cell proliferation and apoptosis analysis

Sections were stained immunohistochemically using anti-BrdU antibody (1∶100, Sigma-Aldrich) and were subjected to TUNEL (Terminal deoxynucleotidyl transferase dUTP nick end labeling), using the ApopTag Plus In Situ Apoptosis Fluorescein Detection Kit in accordance to the manufacture's instruction (Millipore, Billerica, MA).

### In vitro wound healing assay and cell growth curve

For in vitro scratch wound healing assay, the cells were seeded at 1.5×10^5^ cells/well in 24-well plates and were allowed to reach 100% confluence. A scratch wound was created on the cell surface using a micropipette tip. The wound area was photographed by bright-field microscopy at different time points after wounding. The width of the wound was measured and the wound closure rate was calculated. For transwell migration assay, 5×10^4^ cells were seeded in each cell culture insert (BD Falcon Franklin Lakes, NJ), which contains a polyethylene terephthalate membrane (6.5 mm in diameter, 8 µm pore size) and was pre-coated with 10 µg/ml collagen I. Cells were incubated at 37°C for 3 hours. Non-migrated cells were scraped off the upper surface of the membrane with a cotton swab. Migrated cells were fixed by 4% paraformaldehyde and stained with 0.3% crystal violet for photography. The dye in the cells was then dissolved in 10% acetic acid and the absorbance of the dissolved dye was measured at 600 nm. To establish the cell growth curve, the hTCEpi cells were seeded at 2×10^4^ cells/well in 24-well plates and cell numbers were counted thereafter at different time points.

### Western bloting, GST-p38 pull-down and co-immunoprecipitation

The hTCEpi cells were lysed in “egg lysis buffer”, which contains 50 mM Tris pH 7.5, 0.1% NP40, 120 mM NaCl, 1 mM EDTA, 6 mM EGTA, 20 mM NaF, 1 mM Na Pyrophosphate, 30 mM 4-Nitrophenyl phosphate, 1 mM Benzamidine and 1X Protease Inhibitor cocktail (Sigma-Aldrich), and centrifuged at 12000 rpm for 15 min. For Western blotting, the cell lysates were boiled in loading buffer and were applied to electrophoresis on 10% SDS-PAGE. The resolved proteins were transferred to nitrocellulose membranes and detected by Western blotting analyses using antibodies as indicated.

For pull-down assays, the cell lysates were incubated at 4°C for 1 h with GST-p38 fusion protein, followed by incubation with Glutathione-agarose (Sigma-Aldrich) at 4°C overnight. For co-immunoprecipitation assays, the cell lysates were incubated with primary antibodies, followed by incubation with protein A agarose (Invitrogen) at 4°C overnight. After extensive washing, the proteins were eluted from the beads by boiling and subjected to SDS-PAGE and Western blot analyses.

### Statistical analysis

We conducted statistical comparisons using student two-tailed paired *t-*test and ANOVA analyses of variance. We considered values of *p*<0.05 statistically significant.

## Supporting Information

Figure S1 **IKKβ promoted corneal epithelial wound healing is independent of proliferation, apoptosis and macrophage infiltration.** The injured eyes of *Ikkβ^F/F^* and *Ikkβ^ΔCE/ΔCE^* mice were examined by H&E staining for histology and by TUNEL assay to assess apoptosis. The eyes were also examined by immunohistochemistry using anti-BrdU for proliferation and anti-F4/80 for macrophage infiltration (red). Nuclei were identified by DAPI staining (blue). The boundaries of corneal epithelium (epi) and stroma (str) were marked with dotted lines and the staining positive cells were labeled by arrowheads. The picture represents at least 3 slides of each mouse and 2 mice of each genotype used. #: the edge of the wound area.
(TIF)

Figure S2 **IKKβ is required for cytokine promoted wound healing of hTCEpi and HaCaT cells.** (A) hTCEpi and (B, C) HaCaT cells were pre-treated with TPCA-1 (0.5 µM) for 30 min for some experiments, followed by scratch wound healing assay in the presence of various cytokines and growth factors (10 ng/ml). Pictures were taken at 0 and 48 hours after wounding and the wound closure rates were calculated based on mean ± SD of 4 independent experiments. *: *p<*0.05; **: *p<*0.01; ***: *p<*0.001.
(TIF)

Figure S3 **IKKβ was dispensible for the phosphorylation of c-JUN, JNK, ERK, SMAD2/3 in the injured corneal epithelium.** The *Ikkβ^F/F^* and *Ikkβ^ΔCE/ΔCE^* mice were subjected to corneal epithelial debridement injury and the wounded eyes were analyzed by immunohistochemistry for the phosphorylation of c-JUN, JNK, ERK and SMDAD2/3 (red). Nuclei were stained with DAPI (blue). (A) Pictures were taken under fluorescent microscope, and (B) The percentages of phosphor-c-JUN, -JNK, -ERK and SMDAD2/3 positive cell over total corneal epithelial cell in each field were calculated. At least 3 sections of each eye and as least 2 eyes of each genotype and time point were used for calculation and statistical analysis.
(TIF)

## References

[1] Huxford T, Huang DB, Malek S, Ghosh G (1998). The crystal structure of the IkappaBalpha/NF-kappaB complex reveals mechanisms of NF-kappaB inactivation.. Cell.

[2] Rothwarf DM, Zandi E, Natoli G, Karin M (1998). IKK-gamma is an essential regulatory subunit of the IkappaB kinase complex.. Nature.

[3] Hayden MS, Ghosh S (2004). Signaling to NF-kappaB.. Genes Dev.

[4] Schmid JA, Birbach A (2008). IkappaB kinase beta (IKKbeta/IKK2/IKBKB)–a key molecule in signaling to the transcription factor NF-kappaB.. Cytokine Growth Factor Rev.

[5] Li Q, Van Antwerp D, Mercurio F, Lee KF, Verma IM (1999). Severe liver degeneration in mice lacking the IkappaB kinase 2 gene.. Science.

[6] Li ZW, Chu W, Hu Y, Delhase M, Deerinck T (1999). The IKKbeta subunit of IkappaB kinase (IKK) is essential for nuclear factor kappaB activation and prevention of apoptosis.. J Exp Med.

[7] Pasparakis M, Courtois G, Hafner M, Schmidt-Supprian M, Nenci A (2002). TNF-mediated inflammatory skin disease in mice with epidermis-specific deletion of IKK2.. Nature.

[8] Stratis A, Pasparakis M, Markur D, Knaup R, Pofahl R (2006). Localized inflammatory skin disease following inducible ablation of I kappa B kinase 2 in murine epidermis.. J Invest Dermatol.

[9] Bockhart V, Constantin CE, Haussler A, Wijnvoord N, Kanngiesser M (2009). Inhibitor kappaB Kinase beta deficiency in primary nociceptive neurons increases TRP channel sensitivity.. J Neurosci.

[10] Koch KS, Maeda S, He G, Karin M, Leffert HL (2009). Targeted deletion of hepatocyte Ikkbeta confers growth advantages.. Biochem Biophys Res Commun.

[11] Malato Y, Sander LE, Liedtke C, Al-Masaoudi M, Tacke F (2008). Hepatocyte-specific inhibitor-of-kappaB-kinase deletion triggers the innate immune response and promotes earlier cell proliferation during liver regeneration.. Hepatology.

[12] Baxter FO, Came PJ, Abell K, Kedjouar B, Huth M (2006). IKKbeta/2 induces TWEAK and apoptosis in mammary epithelial cells.. Development.

[13] Rasoulpour RJ, Boekelheide K (2007). NF-kappaB activation elicited by ionizing radiation is proapoptotic in testis.. Biol Reprod.

[14] Shibata W, Takaishi S, Muthupalani S, Pritchard DM, Whary MT (2010). Conditional deletion of IkappaB-kinase-beta accelerates helicobacter-dependent gastric apoptosis, proliferation, and preneoplasia.. Gastroenterology.

[15] Chen LW, Chen PH, Chang WJ, Wang JS, Karin M (2007). IKappaB-kinase/nuclear factor-kappaB signaling prevents thermal injury-induced gut damage by inhibiting c-Jun NH2-terminal kinase activation.. Crit Care Med.

[16] Chae S, Eckmann L, Miyamoto Y, Pothoulakis C, Karin M (2006). Epithelial cell I kappa B-kinase beta has an important protective role in Clostridium difficile toxin A-induced mucosal injury.. J Immunol.

[17] Hikoso S, Yamaguchi O, Nakano Y, Takeda T, Omiya S (2009). The I{kappa}B kinase {beta}/nuclear factor {kappa}B signaling pathway protects the heart from hemodynamic stress mediated by the regulation of manganese superoxide dismutase expression.. Circ Res.

[18] Ruocco MG, Maeda S, Park JM, Lawrence T, Hsu LC (2005). I{kappa}B kinase (IKK){beta}, but not IKK{alpha}, is a critical mediator of osteoclast survival and is required for inflammation-induced bone loss.. J Exp Med.

[19] Maeda S, Kamata H, Luo JL, Leffert H, Karin M (2005). IKKbeta couples hepatocyte death to cytokine-driven compensatory proliferation that promotes chemical hepatocarcinogenesis.. Cell.

[20] Zieske JD (2004). Corneal development associated with eyelid opening.. Int J Dev Biol.

[21] Saika S (2007). Yin and yang in cytokine regulation of corneal wound healing: roles of TNF-alpha.. Cornea.

[22] Yu FSX, Yin J, Xu KP, Huang J (2010). Growth factors and corneal epithelial wound healing.. Brain Research Bulletin.

[23] Yamada N, Yanai R, Kawamoto K, Nagano T, Nakamura M (2006). Promotion of corneal epithelial wound healing by a tetrapeptide (SSSR) derived from IGF-1.. Invest Ophthalmol Vis Sci.

[24] Suzuki K, Saito J, Yanai R, Yamada N, Chikama T (2003). Cell-matrix and cell-cell interactions during corneal epithelial wound healing.. Prog Retin Eye Res.

[25] Li Z, Burns AR, Rumbaut RE, Smith CW (2007). gamma delta T cells are necessary for platelet and neutrophil accumulation in limbal vessels and efficient epithelial repair after corneal abrasion.. Am J Pathol.

[26] Hara Y, Matsuura T, Tsukamoto M, Ishizaka S, Saishin M (2001). Effect of tetra-peptide isolated from interleukin 1 (IL-1) on corneal epithelial wound healing in the rabbit.. Exp Eye Res.

[27] Wilson SE, Esposito A (2009). Focus on molecules: interleukin-1: a master regulator of the corneal response to injury.. Exp Eye Res.

[28] Wang X, Kamiyama K, Iguchi I, Kita M, Imanishi J (1994). Enhancement of fibronectin-induced migration of corneal epithelial cells by cytokines.. Invest Ophthalmol Vis Sci.

[29] Chikama T, Hayashi Y, Liu CY, Terai N, Terai K (2005). Characterization of tetracycline-inducible bitransgenic Krt12rtTA/+/tet-O-LacZ mice.. Invest Ophthalmol Vis Sci.

[30] Imanishi J, Kamiyama K, Iguchi I, Kita M, Sotozono C (2000). Growth factors: importance in wound healing and maintenance of transparency of the cornea.. Prog Retin Eye Res.

[31] Klenkler B, Sheardown H (2004). Growth factors in the anterior segment: role in tissue maintenance, wound healing and ocular pathology.. Exp Eye Res.

[32] Jester JV, Huang J, Fisher S, Spiekerman J, Chang JH (2003). Myofibroblast differentiation of normal human keratocytes and hTERT, extended-life human corneal fibroblasts.. Invest Ophthalmol Vis Sci.

[33] Saika S, Okada Y, Miyamoto T, Yamanaka O, Ohnishi Y (2004). Role of p38 MAP kinase in regulation of cell migration and proliferation in healing corneal epithelium.. Invest Ophthalmol Vis Sci.

[34] Mazie AR, Spix JK, Block ER, Achebe HB, Klarlund JK (2006). Epithelial cell motility is triggered by activation of the EGF receptor through phosphatidic acid signaling.. J Cell Sci.

[35] Kimura K, Teranishi S, Yamauchi J, Nishida T (2008). Role of JNK-dependent serine phosphorylation of paxillin in migration of corneal epithelial cells during wound closure.. Invest Ophthalmol Vis Sci.

[36] Hutcheon AE, Guo XQ, Stepp MA, Simon KJ, Weinreb PH (2005). Effect of wound type on Smad 2 and 4 translocation.. Invest Ophthalmol Vis Sci.

[37] Sastre AP, Grossmann S, Reusch HP, Schaefer M (2008). Requirement of an intermediate gene expression for biphasic ERK1/2 activation in thrombin-stimulated vascular smooth muscle cells.. J Biol Chem.

[38] Zarubin T, Han J (2005). Activation and signaling of the p38 MAP kinase pathway.. Cell Res.

[39] Tsai PW, Shiah SG, Lin MT, Wu CW, Kuo ML (2003). Up-regulation of vascular endothelial growth factor C in breast cancer cells by heregulin-beta 1. A critical role of p38/nuclear factor-kappa B signaling pathway.. J Biol Chem.

[40] Eckmann L, Nebelsiek T, Fingerle AA, Dann SM, Mages J (2008). Opposing functions of IKKbeta during acute and chronic intestinal inflammation.. Proc Natl Acad Sci U S A.

[41] Hori J (2008). Mechanisms of immune privilege in the anterior segment of the eye: what we learn from corneal transplantation.. J Ocul Biol Dis Infor.

[42] Lee S, Andrieu C, Saltel F, Destaing O, Auclair J (2004). IkappaB kinase beta phosphorylates Dok1 serines in response to TNF, IL-1, or gamma radiation.. Proc Natl Acad Sci U S A.

[43] Chandrasekar B, Mummidi S, Mahimainathan L, Patel DN, Bailey SR (2006). Interleukin-18-induced human coronary artery smooth muscle cell migration is dependent on NF-kappaB- and AP-1-mediated matrix metalloproteinase-9 expression and is inhibited by atorvastatin.. J Biol Chem.

[44] Wu Y, Deng J, Rychahou PG, Qiu S, Evers BM (2009). Stabilization of snail by NF-kappaB is required for inflammation-induced cell migration and invasion.. Cancer Cell.

[45] Xie W, Wang Y, Huang Y, Yang H, Wang J (2009). Toll-like receptor 2 mediates invasion via activating NF-kappaB in MDA-MB-231 breast cancer cells.. Biochem Biophys Res Commun.

[46] Shuto T, Xu H, Wang B, Han J, Kai H (2001). Activation of NF-kappa B by nontypeable Hemophilus influenzae is mediated by toll-like receptor 2-TAK1-dependent NIK-IKK alpha/beta-I kappa B alpha and MKK3/6-p38 MAP kinase signaling pathways in epithelial cells.. Proc Natl Acad Sci U S A.

[47] Wang C, Deng L, Hong M, Akkaraju GR, Inoue J (2001). TAK1 is a ubiquitin-dependent kinase of MKK and IKK.. Nature.

[48] Lee J, Mira-Arbibe L, Ulevitch RJ (2000). TAK1 regulates multiple protein kinase cascades activated by bacterial lipopolysaccharide.. J Leukoc Biol.

[49] Qiao H, Shibaki A, Long HA, Wang G, Li Q (2009). Collagen XVII participates in keratinocyte adhesion to collagen IV, and in p38MAPK-dependent migration and cell signaling.. J Invest Dermatol.

[50] Harper EG, Alvares SM, Carter WG (2005). Wounding activates p38 map kinase and activation transcription factor 3 in leading keratinocytes.. J Cell Sci.

[51] Klekotka PA, Santoro SA, Zutter MM (2001). alpha 2 integrin subunit cytoplasmic domain-dependent cellular migration requires p38 MAPK.. J Biol Chem.

[52] Yanai R, Yamada N, Inui M, Nishida T (2006). Correlation of proliferative and anti-apoptotic effects of HGF, insulin, IGF-1, IGF-2, and EGF in SV40-transformed human corneal epithelial cells.. Exp Eye Res.

[53] Yin MJ, Yamamoto Y, Gaynor RB (1998). The anti-inflammatory agents aspirin and salicylate inhibit the activity of I(kappa)B kinase-beta.. Nature.

[54] Busti AJ, Hooper JS, Amaya CJ, Kazi S (2005). Effects of perioperative antiinflammatory and immunomodulating therapy on surgical wound healing.. Pharmacotherapy.

[55] Schalnus R (2003). Topical nonsteroidal anti-inflammatory therapy in ophthalmology.. Ophthalmologica.

[56] Anstead GM (1998). Steroids, retinoids, and wound healing.. Adv Wound Care.

[57] Hengge UR, Ruzicka T, Schwartz RA, Cork MJ (2006). Adverse effects of topical glucocorticosteroids.. J Am Acad Dermatol.

[58] Liu CY, Zhu G, Converse R, Kao CW, Nakamura H (1994). Characterization and chromosomal localization of the cornea-specific murine keratin gene Krt1.12.. J Biol Chem.

[59] Perl AK, Wert SE, Nagy A, Lobe CG, Whitsett JA (2002). Early restriction of peripheral and proximal cell lineages during formation of the lung.. Proc Natl Acad Sci U S A.

[60] Li ZW, Omori SA, Labuda T, Karin M, Rickert RC (2003). IKK beta is required for peripheral B cell survival and proliferation.. J Immunol.

[61] Deng M, Chen WL, Takatori A, Peng Z, Zhang L (2006). A role for the mitogen-activated protein kinase kinase kinase 1 in epithelial wound healing.. Mol Biol Cell.

