# Supplementary figures and images for: IκB Kinase β Regulates Epithelium Migration during Corneal Wound Healing
Source: PLoS One. 2011 Jan 17;6(1):e16132. doi: 10.1371/journal.pone.0016132 (PMC3022035; doi:10.1371/journal.pone.0016132)

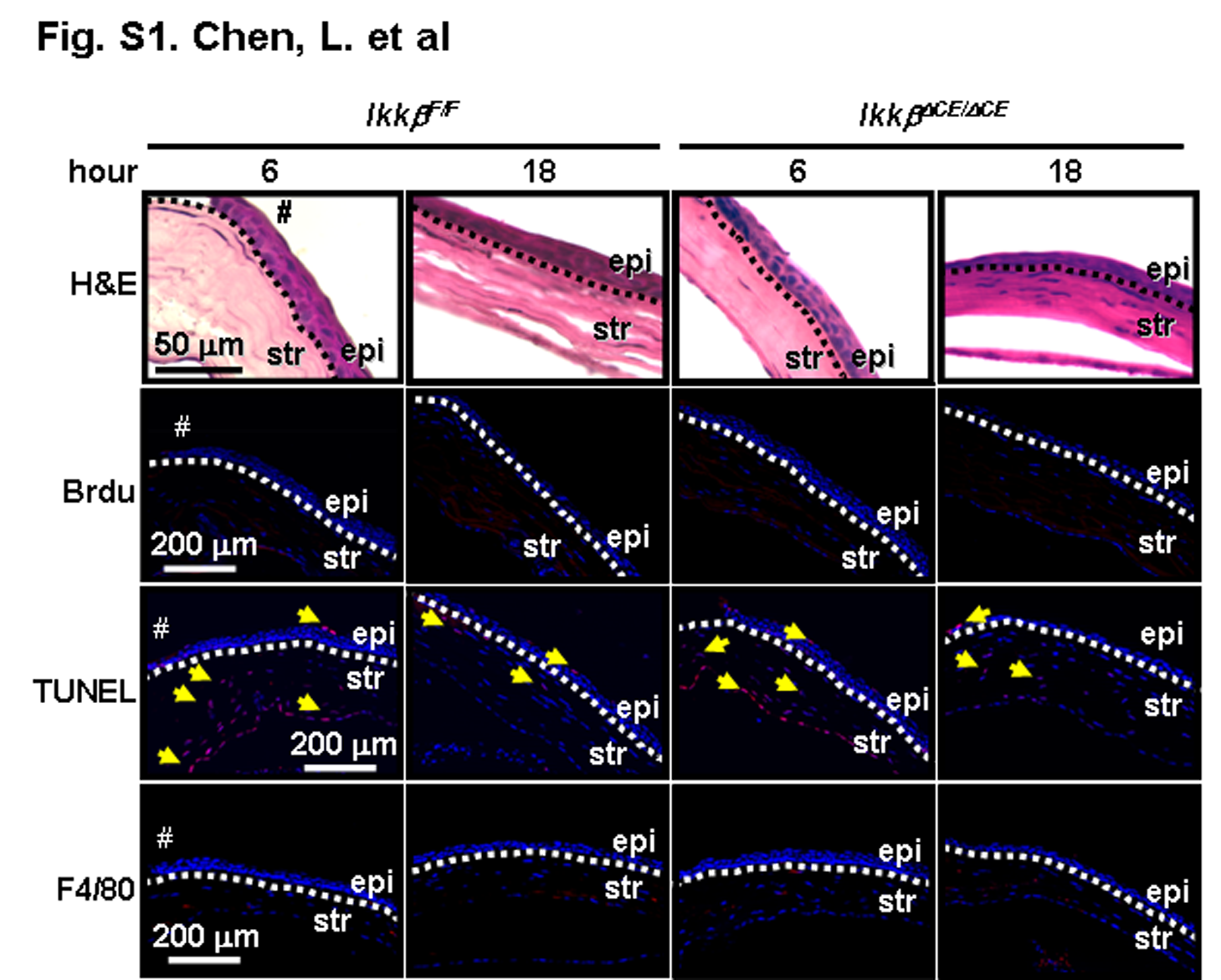

Supplement: Figure S1 — IKKβ promoted corneal epithelial wound healing is independent of proliferation, apoptosis and macrophage infiltration. The injured eyes of IkkβF/F and IkkβΔCE/ΔCE mice were examined by H&E staining for histology and by TUNEL assay to assess apoptosis. The eyes were also examined by immunohistochemistry using anti-BrdU for proliferation and anti-F4/80 for macrophage infiltration (red). Nuclei were identified by DAPI staining (blue). The boundaries of corneal epithelium (epi) and stroma (str) were marked with dotted lines and the staining positive cells were labeled by arrowheads. The picture represents at least 3 slides of each mouse and 2 mice of each genotype used. #: the edge of the wound area. (TIF) [file pone.0016132.s001.tif]

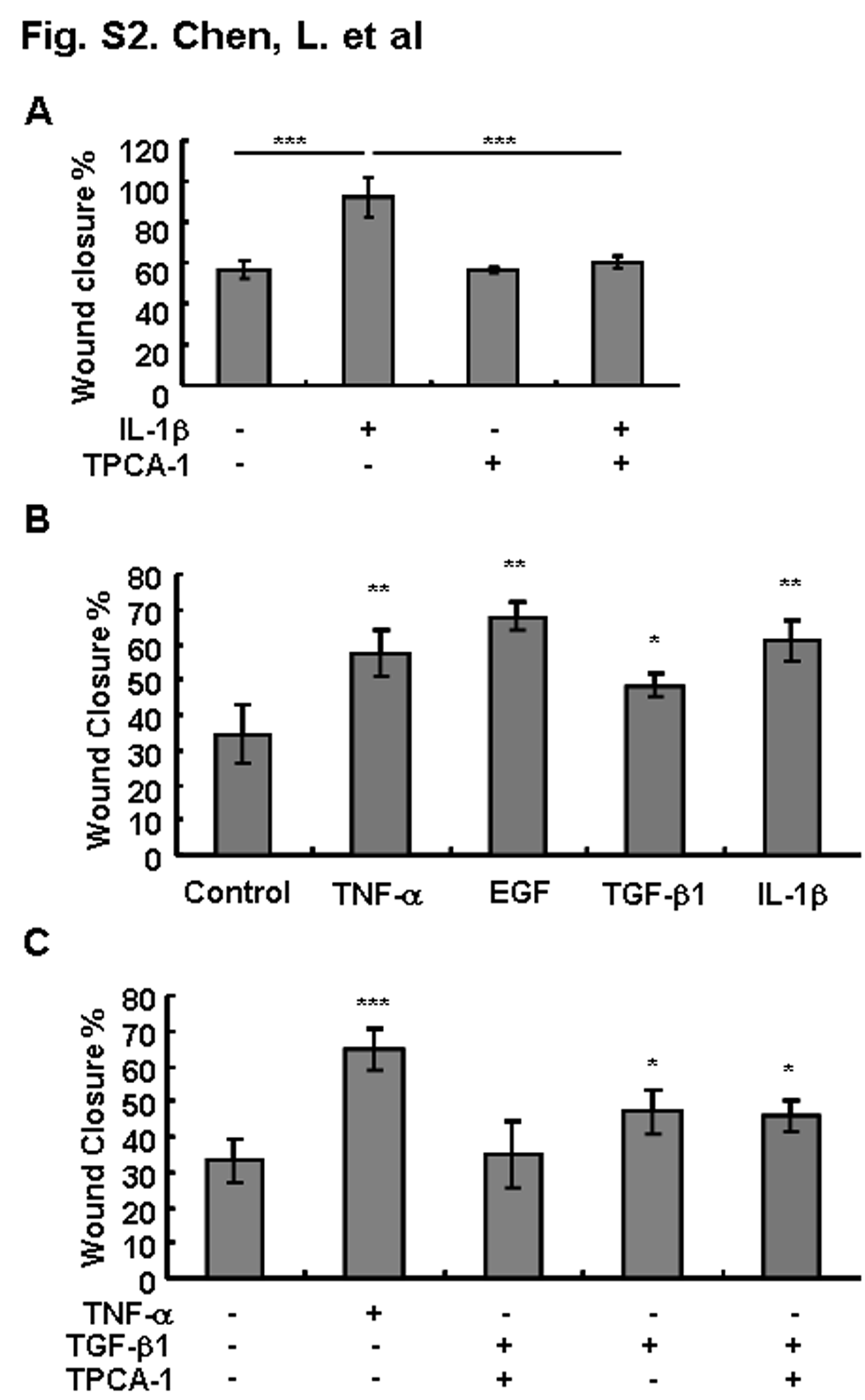

Supplement: Figure S2 — IKKβ is required for cytokine promoted wound healing of hTCEpi and HaCaT cells. (A) hTCEpi and (B, C) HaCaT cells were pre-treated with TPCA-1 (0.5 µM) for 30 min for some experiments, followed by scratch wound healing assay in the presence of various cytokines and growth factors (10 ng/ml). Pictures were taken at 0 and 48 hours after wounding and the wound closure rates were calculated based on mean ± SD of 4 independent experiments. *: p<0.05; **: p<0.01; ***: p<0.001. (TIF) [file pone.0016132.s002.tif]

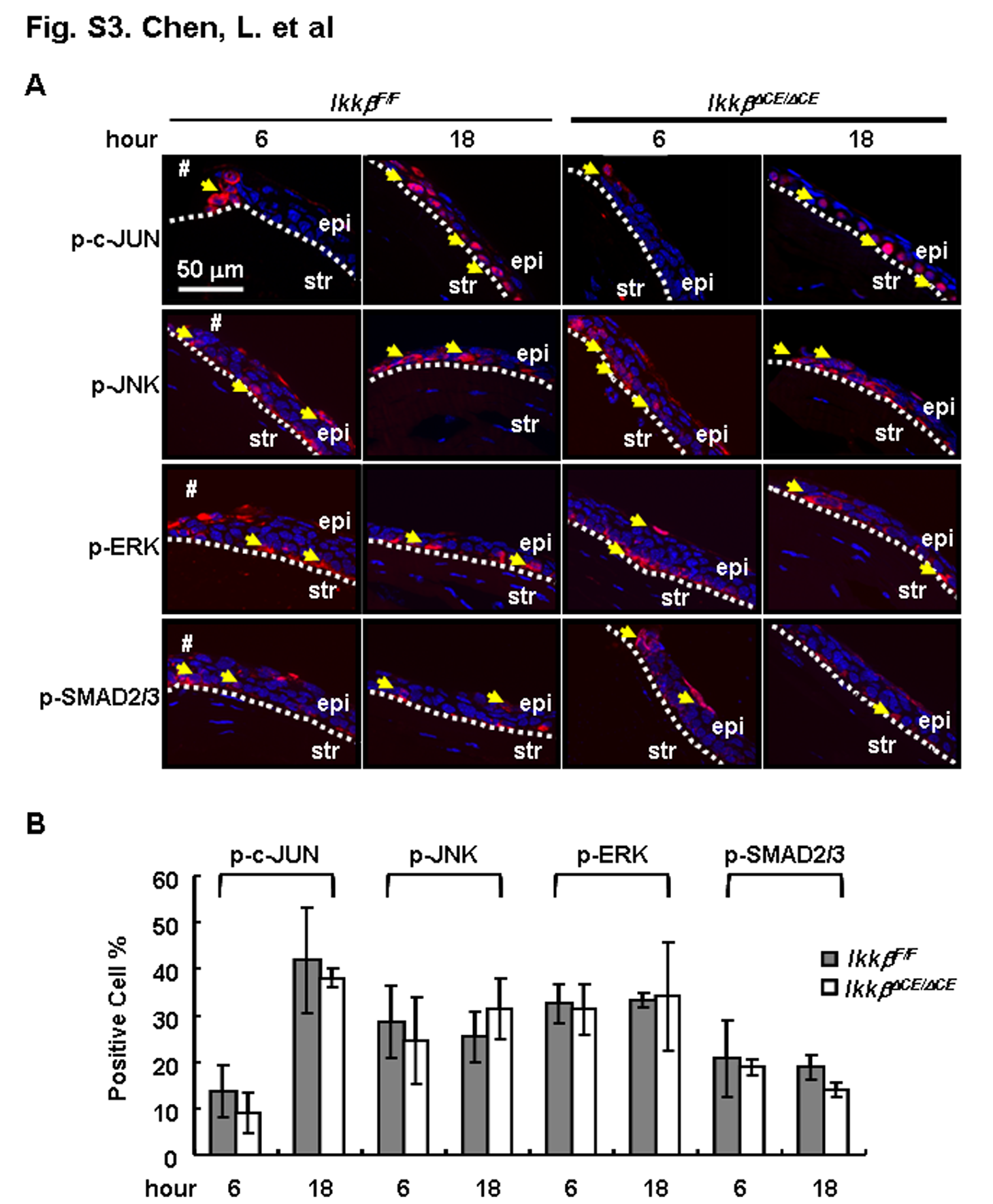

Supplement: Figure S3 — IKKβ was dispensible for the phosphorylation of c-JUN, JNK, ERK, SMAD2/3 in the injured corneal epithelium. The IkkβF/F and IkkβΔCE/ΔCE mice were subjected to corneal epithelial debridement injury and the wounded eyes were analyzed by immunohistochemistry for the phosphorylation of c-JUN, JNK, ERK and SMDAD2/3 (red). Nuclei were stained with DAPI (blue). (A) Pictures were taken under fluorescent microscope, and (B) The percentages of phosphor-c-JUN, -JNK, -ERK and SMDAD2/3 positive cell over total corneal epithelial cell in each field were calculated. At least 3 sections of each eye and as least 2 eyes of each genotype and time point were used for calculation and statistical analysis. (TIF) [file pone.0016132.s003.tif]
